# Circulating nucleosomes as predictive markers of severe acute pancreatitis

**DOI:** 10.1186/s40560-016-0135-6

**Published:** 2016-02-17

**Authors:** Anne K. Penttilä, Ari Rouhiainen, Leena Kylänpää, Harri Mustonen, Pauli Puolakkainen, Heikki Rauvala, Heikki Repo

**Affiliations:** Department of Surgery, Helsinki University Hospital, P.O. Box 340, 00029 HUS Helsinki, Finland; Neuroscience Center, University of Helsinki, P.O. Box 56, 00014 Helsinki, Finland; Department of Biosciences, University of Helsinki, P.O. Box 65, 00014 Helsinki, Finland; Institute of Clinical Medicine, University of Helsinki, P.O. Box 340, 00029 HUS Helsinki, Finland; Department of Bacteriology and Immunology, University of Helsinki, The Haartman Institute, P.O. Box 21, 00014 Helsinki, Finland

**Keywords:** Biomarkers, Cellular damage, Nucleosomes, Organ dysfunction, Pancreatitis

## Abstract

**Background:**

The components of nucleosomes, which contain DNA and histones, are released into the circulation from damaged cells and can promote inflammation. We studied whether the on-admission levels of circulating nucleosomes predict the development of severe acute pancreatitis (AP), in particular among the patients who present without clinical signs of organ dysfunction.

**Methods:**

This is a prospective study of 74 AP patients admitted to Helsinki University Hospital from 2003 to 2007. Twenty-three patients had mild, 27 moderately severe, and 24 severe AP as defined by the revised Atlanta criteria. 14/24 severe AP patients had no sign of organ dysfunction on admission (modified marshall score <2). Blood samples were obtained on admission and the plasma levels of nucleosomes were measured using enzyme-linked immunosorbent assay.

**Results:**

The on-admission levels of nucleosomes were significantly higher in severe AP than in mild or moderately severe AP (*p* < 0.001 for all), higher in non-survivors (*n* = 8) than in survivors (p = 0.019), and correlated with the on-admission levels of C-reactive protein (*p* < 0.001) and creatinine (*p* < 0.001). Among the AP patients who presented without organ dysfunction, the on-admission nucleosome level was an independent predictor of severe AP (*p* = 0.038, gender-adjusted forward-stepping logistic regression).

**Conclusions:**

Circulating nucleosome levels may be helpful in identifying, on admission to hospital, the AP patients who present without clinical signs of organ dysfunction, and, yet, are bound to develop organ dysfunction during hospitalization.

## Background

Acute pancreatitis (AP) is usually a mild disease with favorable outcome. However, about 20 % of the patients develop moderately severe or severe disease, as defined by the revised Atlanta classification [1]. Moderately severe AP is characterized by the presence of local complications and/or transient (<48 h) organ dysfunction (OD) and very low mortality [2]. In severe AP, OD is persistent and mortality high, up to 70 % [2–5]. Evidence has accumulated to show that early aggressive intravenous hydration decreases morbidity and mortality [6, 7]. In addition, the patients at risk to develop severe AP, particularly those who present without OD, might benefit from immunomodulatory treatment [8–10]. About half of the AP patients with OD do not have clinical signs of OD at presentation [8, 11, 12]. At present, there are no means to identify these patients on admission to the hospital.

The inflammatory reaction in AP is considered to have its origin in premature activation of pancreatic proteases promoting acinar cell apoptosis and necrosis. Damaged or dying pancreatic acinar cells release intracellular contents including nuclear damage-associated molecular patterns (nDAMPs), such as DNA and histones, which promote the accumulation of innate immune cells into the pancreas and generation of cytokines, among other soluble mediators of inflammation. The release of phlogistic mediators into the circulation elicits systemic inflammation, which is considered to contribute to the development of remote organ injury (for reviews, see refs [13, 14]).

Nucleosome, a subunit of nuclear chromatin, consists of a central core protein formed by an octamer of the double-represented histone and 147 base pairs of double-stranded DNA [15]. Cellular damage, such as apoptosis and necrosis, promotes the release of nucleosomes, among other nDAMPS, into the extracellular space, where DNA and histone exhibit pro-inflammatory activity [14, 16]. Nucleosomes can also be exported within neutrophil extracellular traps (NETs) during NETosis, a unique form of neutrophil cell death at sites of infection and inflammation [17, 18]. Although elevated levels of circulating nucleosomes are detected in patients with sepsis [19, 20], in other disorders characterized of systemic inflammation [21–23], and in experimental AP [24], to our knowledge, nucleosome levels have not been systematically studied in patients with AP. This prompted us to investigate whether the on-admission plasma nucleosome levels associate with the severity of AP and predict the development of severe AP, in other words persistent OD.

## Methods

### Patients

A cohort of 74 prospectively collected non-consecutive patients with AP admitted to Helsinki University Hospital between June 2003 and December 2007 were included in the study. Exclusion criteria were previous history of chronic pancreatitis and the onset of symptoms more than 72 h before admittance to the hospital.

The diagnosis of AP was made if two of the following three features were present: acute onset of upper epigastric pain, serum or plasma amylase level at least three times greater than the upper limit of normal, and characteristic findings of AP in imaging studies (computed tomography or magnetic resonance imaging). The patients were treated according to the international guidelines [25] with, e.g., early aggressive intravenous hydration, no routine use of prophylactic antibiotics, nasojejunal tube for enteral feeding in severe AP, and endoscopic retrograde cholangiopancreatography if concurrent cholangitis was present.

**Fig. 1:**
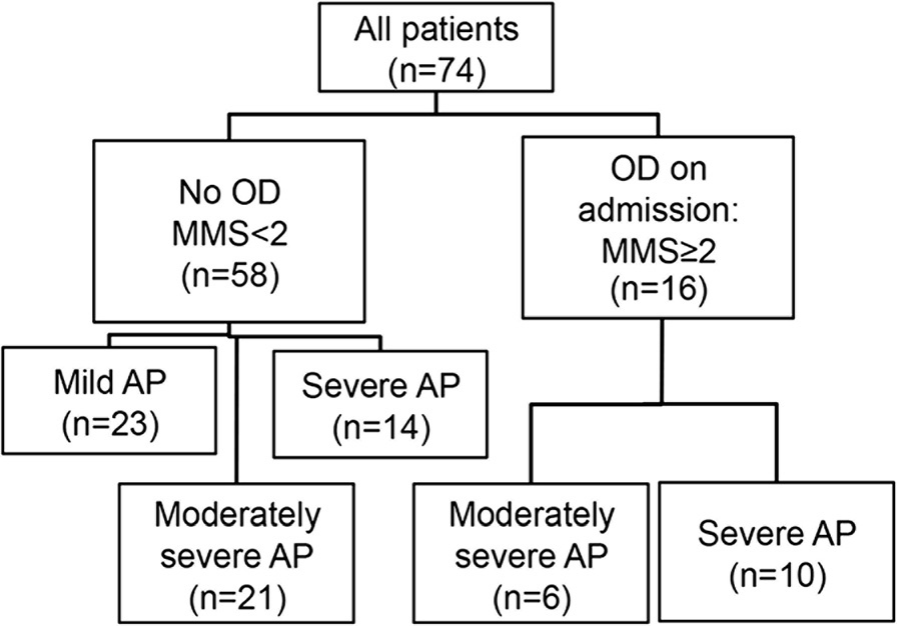
Flow chart of the patients. Patients’ classification according to admission modified marshall score (MMS) and patients’ outcome according to the revised Atlanta criteria [1]. *OD* organ dysfunction, *AP* acute pancreatitis

After inclusion, demographic and clinical characteristics of patients were collected from medical charts. The severity of AP was graded retrospectively according to the revised Atlanta classification [1] into mild (no systemic or local complication), moderately severe (local and/or systemic complication without persistent OD), and severe (persistent OD). Acute physiology and chronic health evaluation (APACHE) II score, sepsis-related organ failure assessment (SOFA) score, and modified marshall score (MMS) were determined to evaluate the severity of OD on admission. MMS [26] was used for assessing the presence of OD on admission, as recommended in the revised Atlanta classification [1]. In MMS, three organ systems (respiratory, renal, and cardiac) are assessed, and if the score is ≥2 for one of those organ systems, OD is present. The flow chart of the patients is presented in Fig. 1.

Each patient, or next to kin, gave informed consent. Ethics Committee of Helsinki University Hospital (Department of Surgery) approved the study.

### Samples and sample analyses

The plasma samples were taken 0–12 h after admission, collected into EDTA-treated tubes and stored at −80 °C until they were assayed. Nucleosomes were quantified with Cell Death Detection ELISA^PLUS^ (Roche, Basel, Switzerland) according to the instructions of the manufacturer. The results are presented as absorbance units (AU). Negative values were computed to zero.

Plasma levels of C-reactive protein (CRP) (normal reference range less than 10 mg/L) and creatinine (normal reference range 50–90 μmol/L) were determined in accordance with the hospital laboratory routine. CRP and creatinine levels were used as reference markers because they belong to routine follow-up blood chemistry of AP patients and have prognostic value in AP [7, 34].

The median storage time of the plasma samples was long, in mild AP group 9.3 years (range 6.2–9.8 years), moderately severe AP group 8.3 years (range 5.8–9.8 years), and severe AP group 7.3 years (range 5.3–9.8 years), (*p* < 0.001, Jonckheere-Terpstra test for trend). However, the sample age did not correlate with nucleosome level in mild, moderately severe or severe AP (*p* = 0.153, *p* = 0.928, and *p* = 0.631, respectively), and in multivariate logistic regression analysis nucleosome level remained as an independent predictor of OD regardless of the storage time.

### Statistics

Statistical analysis was performed using IBM SPSS® Statistic version 19 (SPSS, Chicago, Illinois, USA) statistical software. Nonparametric tests were used because of the skewness of the data. The results are given as medians and interquartile ranges (IQRs) or number of patients and percentages. Comparisons between two groups were made using the Mann-Whitney *U* test for continuous variables or using Fisher’s exact test for binary variables. Comparisons between three ordered groups were tested with the Jonckheere-Terpstra test for trend. Correlations between two continuous variables were done using Spearman rank correlation. *P* values of less than 0.05 were considered significant, and double-sided tests were used. Receiver operator characteristic (ROC) curve analysis was used to find a clinically optimal cutoff value for each biomarker. In this study, we determined the specificity >90 % and chose the point on the curve where the longest increase in the sensitivity of the slope declines. Areas under the ROC curves (AUC) were calculated, as well as corresponding sensitivities, specificities, positive likelihood ratios (+LR), negative likelihood ratios (−LR), and diagnostic odds ratios (DOR) for cutoff values, with 95 % confidence intervals [27]. DOR is the ratio of the odds of positive test result among patients with OD to the odds of a positive test result among the patients without OD. The higher the value, the better the discriminatory test performance is [28]. Finally, logistic regression analysis was performed to identify independent markers predicting severe AP. Forward conditional stepping was used to select variables into the post hoc model with *p* < 0.05 inclusion criteria. Interactions were considered, but no significant interactions were found.

## Results

### Patients

Characteristics of the patients are shown in Table 1. All patients with severe AP developed either respiratory or renal failure needing mechanical invasive ventilation and/or haemodialysis. Seven of them (29 %) died, four of whom during the first hospital week (range 1–6 days), and the other three patients 11–90 days after admission. One patient, already recovering from moderately severe AP, experienced sudden death of unknown immediate cause.

**Table 1:** Baseline characteristics of patients

Variable	Mild	Moderately severe	Severe
	(*n* = 23)	(*n* = 27)	(*n* = 24)
Male sex, (%)	15 (65)	19 (70)	23 (96)
Age (years)	44 (37–64)	52 (44–61)	43 (37–51)
Etiology of acute pancreatitis, (%)			
Alcohol	13 (57)	20 (74)	21 (88)
Biliary	6 (26)	7 (26)	1 (4)
Idiopathic or other	4 (17)	0	2 (8)
Duration of symptoms (hr)	24 (12–60)	24 (12–48)	24 (12–48)
CRP on admission (mg/L)	8 (5–17)	84 (9–231)	109 (15–292)
Creatinine on admission (μmol/L)	59 (49–68)	61 (53–101)	92 (66–243)
APACHE II	6 (1–7)	8 (5–10)	8 (6–15)
SOFA on admission	0 (0–1)	1 (1–3)	4 (1–6)
MMS on admission	0 (0–1)	1 (0–4)	1 (1–4)
MMS < 2 on admission, (%)	23 (100)	21 (78)	14 (58)
MMS ≥ 2 on admission, (%)	0	6 (22)	10 (42)
Mechanical invasive ventilation, (%)	0	1 (4)	22 (92)
Haemodialysis, (%)	0	0	16 (67)
Length of hospital stay (days)	4 (3–6)	11 (9–15)	28 (17–35)
Mortality, (%)	0	1 (4)	7 (29)

All numerical data are median (interquartile range) or number (%). APACHE II was determined using the most abnormal value for each physiological variable within 24 h of admission to the hospital

*APACHE* acute physiology and chronic health evaluation, *CRP* C-reactive protein, *MMS* modified marshall score, *SOFA* sepsis-related organ failure assessment score

### On-admission nucleosome levels correlate with severity of AP

The on-admission levels of nucleosomes increased along with the severity of AP and were significantly higher in patients with severe AP than in mild or moderately severe AP (*p* < 0.001) and higher in non-survivors than in survivors (*p* = 0.019, Table 2).

**Table 2:** The levels of circulating nucleosomes on admission in relation to the severity of acute pancreatitis and among survivors and non-survivors

	Nucleosome level (AU)
Mild	0.005 (0.002–0.14)
Moderately severe	0.09 (0.03–0.32)
Severe	0.30 (0.18–0.45)
	*p* value <0.001^a^
Survivors	0.09 (0.02–0.27)
Non-survivors	0.38 (0.19–0.77)
	*p* value = 0.019^b^

All numerical data are median (interquartile range)

*AU* absorbance unit

^a^Jonckheere-Terpstra for trend

^b^Mann-Whitney *U* test

The nucleosome level correlated with MMS (*r* = 0.525, *p* < 0.001), APACHE II (*r* = 0.414, *p* < 0.001), and SOFA score (*r* = 0.392, *p* = 0.001). There was also a positive correlation between on-admission levels of nucleosomes and CRP (*r* = 0.422, *p* < 0.001), between those of nucleosomes and creatinine (*r* = 0.423, *p* < 0.001), and those of CRP and creatinine (*r* = 0.396, *p* < 0.001).

### Nucleosome, CRP, and creatinine levels as predictors of severe AP

#### All patients

Classification criteria, the Atlanta criteria, and their revised form [1] have been commonly used also in predicting the outcome of AP. We therefore first determined if the on-admission level of circulating nucleosomes predicts the development of severe AP among all patients categorized according to the revised Atlanta criteria [1].

**Fig. 2:**
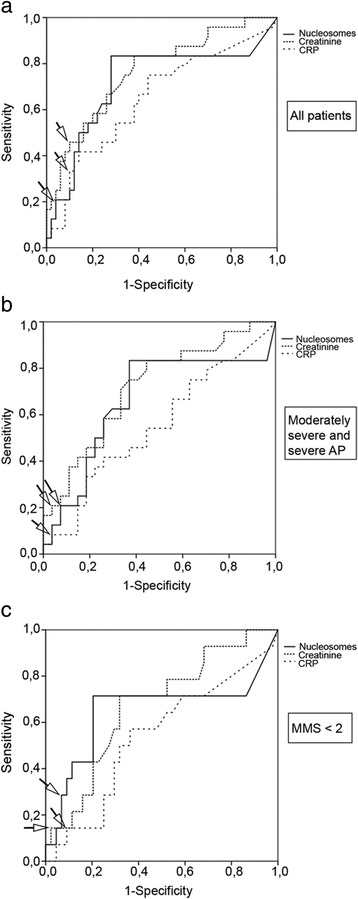
Receiver-operating characteristic curves of circulating nucleosomes, C-reactive protein (CRP) and creatinine for the prediction of severe acute pancreatitis among **a** all patients (*n* = 74), **b** patients with moderately severe or severe acute pancreatitis (*n* = 51), and **c** patients with modified marshall score <2 on admission (*n* = 58). *Arrows* point to clinically optimal cutoff points used to calculate the statistical parameters of each biomarker for Table 4

The predictive value was measured by determining the AUCs from the ROC curve (Fig. 2a). AUCs were 0.718 for nucleosomes, 0.770 for creatinine, and 0.673 for CRP (Table 4), indicating that the predictive values of the variables were comparable. We then chose clinically optimal cutoff values (specificity >90 %) from the ROC curves to analyze the corresponding statistical parameters for the biomarkers to predict severe AP. Of the three variables studied, creatinine (cutoff ≥110 μmol/L) had the highest predictive power of OD with the sensitivity of 46 %, the specificity of 91 % (Table 4).

In univariate logistic regression analysis nucleosomes, CRP and creatinine, both as continuous and binary variables, predicted the development of severe AP. The stepwise forward logistic regression analysis of CRP, creatinine, and nucleosomes (gender adjusted) revealed that creatinine, as a binary variable (cutoff ≥110 μmol/L), was an independent, significant predictor of severe AP (Table 5).

#### Moderately severe AP and severe AP

Because most patients with mild AP recover uneventfully within a few days, we excluded these patients to reveal whether moderately severe AP (*n* = 27) can be distinguished from severe AP (*n* = 24) using the on-admission level of circulating nucleosomes.

AUCs were 0.661 for nucleosomes, 0.717 for creatinine, and 0.550 for CRP (Table 4). We then chose the new clinically optimal cutoff values to predict severe AP with high specificity (>90 %) optimized for moderately severe and severe AP patients from the ROC curves (Fig. 2b). The cutoff points were ≥386 mg/L for CRP, ≥287 μmol/L for creatinine, and ≥0.57 AU for nucleosomes. The specificity and sensitivity for nucleosomes and those for creatinine were comparable (Table 4).

In univariate logistic regression analysis, however, only male gender was a significant predictor of severe AP. Using the gender-adjusted stepwise forward logistic regression analysis of nucleosomes and creatinine, only male gender was an independent predictor of severe AP (Table 5).

#### Predicting severe AP in patients with OD on admission (*n* = 16)

A question of clinical interest is whether nucleosome levels distinguish, on admission, transient OD patients from persistent OD patients. Six of the 16 patients who presented with OD had transient OD, in other words, OD resolved within 48 h, and were ultimately allocated into the moderately severe AP group. The nucleosome, CRP, or creatinine levels of the six transient OD patients did not differ significantly from those of the ten persistent OD patients (Table 3).

**Table 3:** Admission levels of circulating nucleosomes, C-reactive protein, and creatinine of the patients who presented with organ dysfunction (MMS ≥ 2)

Variable	Transient OD (*n* = 6)	Persistent OD (*n* = 10)	*p* value^a^
Nucleosomes (AU)	0.41 (0.18–0.71)	0.39 (0.24–0.75)	0.635
CRP (mg/L)	234 (79–325)	297 (222–340)	0.562
Creatinine (μmol/L)	163 (60–291)	269 (156-390)	0.118

All numerical data are median (interquartile range)

*AU* absorbance unit, *CRP* C-reactive protein, *MMS* modified marshall score, *OD* organ dysfunction

^a^Mann-Whitney *U* test

#### Nucleosome levels predict severe AP among patients without OD on admission (*n* = 58)

A total of 14/24 patients with severe AP and another 44 patients with mild or moderately severe AP had MMS <2 on admission (Fig. 1). Thus, we analyzed if the variable studied predicted the development of OD of the 14 patients who presented without OD. The AUCs were 0.648 for nucleosome, 0.670 for creatinine, and 0.539 for CRP (Table 4). We then determined the clinically optimal cutoff values (specificity >90 %) using the ROC curves of the 58 patients without OD on admission (Fig. 2c). The new cutoff values were much alike compared to the cutoff values of all the patients (Table 4).

**Table 4:** Performance of circulating nucleosomes, C-reactive protein, and creatinine in predicting severe acute pancreatitis

	Cutoff	AUC	Sensitivity	Specificity	+LR	−LR	DOR
All patients (*n* = 74)							
Nucleosomes	≥0.57 AU	0.718	0.21	0.96	5.2	0.83	6.3
		(0.578–0.858)	(0.09–0.40)	(0.87–0.99)	(1.1–24.9)	(0.67–1.02)	(1.1–35.4)
CRP	≥264 mg/L	0.673	0.33	0.91	3.8	0.73	5.2
		(0.583–0.807)	(0.18–0.53)	(0.81–0.96)	(1.4–10.4)	(0.55–0.98)	(1.5–18.1)
Creatinine	≥110 μmol/L	0.770	0.46	0.91	5.2	0.59	8.8
		(0.654–0.886)	(0.28–0.65)	(0.81–0.96)	(2.0–13.4)	(0.41–0.87)	(2.6–29.8)
Moderately severe and severe AP patients (*n* = 51)							
Nucleosomes	≥0.57 AU	0.661	0.21	0.95	2.8	0.86	3.3
		(0.502–0.821)	(0.09–0.40)	(0.77–0.98)	(0.6–13.2)	(0.68–1.08)	(0.6–18.8)
CRP	≥386 mg/L	0.550	0.08	0.96	2.3	0.95	2.4
		(0.390–0.710)	(0.02–0.26)	(0.82–0.99)	(0.2–23.3)	(0.83–1.10)	(0.2–27.9)
Creatinine	≥287 μmol/L	0.717	0.21	0.96	5.6	0.82	6.8
		(0.576–0.858)	(0.09–0.41)	(0.82–0.99)	(0.71–44.8)	(0.66–1.02)	(0.7–63.4)
Patients with modified marshall score <2 on admission (*n* = 58)							
Nucleosomes	≥0.39 AU	0.648	0.29	0.93	4.2	0.77	5.5
		(0.443–0.852)	(0.12–0.55)	(0.82–0.98)	(1.1–16.5)	(0.55–1.08)	(1.1–28.4)
CRP	≥227 mg/L	0.539	0.14	0.92	1.8	0.93	2.0
		(0.362–0.716)	(0.04–0.40)	(0.82–0.97)	(0.37–8.9)	(0.74–1.17)	(0.3–12.0)
Creatinine	≥139 μmol/L	0.670	0.14	0.98	7.3	0.87	8.3
		(0.512–0.829)	(0.04–0.40)	(0.90–1.0)	(0.7–74.6)	(0.70–1.09)	(0.7–99.7)

95 % confidence intervals are given in parentheses

*AU* absorbance unit, *AUC* area under the curve, *CRP* C-reactive protein, *DOR* diagnostic odds ratio, *LR* likelihood ratio

In univariate logistic regression analysis, only nucleosome, as a continuous or a binary variable, was a significant predictor of severe AP. Using the gender adjusted stepwise forward logistic regression analysis model of nucleosomes and creatinine, nucleosome as a continuous variable served as an independent predictor of severe AP (Table 5).

**Table 5:** Univariate and multivariate analysis of circulating nucleosomes, C-reactive protein, and creatinine in predicting severe acute pancreatitis

	OR	95 % CI	*p* value
All patients (*n* = 74)			
Univariate analysis			
Age (year)	0.981	0.948–1.015	0.273
Male gender	10.824	1.341–87.370	0.025
Nucleosomes (AU)	15.086	1.785–127.501	0.0127
CRP (mg/L)	1.004	1.000–1.008	0.045
Creatinine (μmol/L)	1.010	1.003–1.017	0.005
Nucleosomes ≥0.57 AU	6.316	1.127–35.402	0.036
CRP ≥264 mg/L	4.500	1.283–15.778	0.019
Creatinine ≥110 μmol/L	7.615	2.239–25.900	0.001
Multivariate analysis			
Male gender	8.204	0.968–69.511	0.054
Creatinine ≥110 μmol/L	6.180	1.742–21.925	0.005
Patients with moderately severe or severe acute pancreatitis (*n* = 51)			
Univariate analysis			
Age (year)	0.974	0.936–1.014	0.207
Male gender	9.684	1.110–84.465	0.040
Nucleosomes (AU)	5.602	0.739–42.474	0.095
CRP (mg/L)	1.001	0.997–1.005	0.579
Creatinine (μmol/L)	1.007	1.000–1.013	0.052
Nucleosomes ≥0.57 AU	3.289	0.575–18.834	0.181
CRP ≥386 mg/L	2.364	0.201–27.852	0.494
Creatinine ≥287 μmol/L	6.842	0.738–63.442	0.091
Multivariate analysis			
Male gender	9.684	1.110–84.465	0.040
Patients with modified marshall score <2 on admission (*n* = 58)			
Univariate analysis			
Age (year)	0.976	0.936–1.017	0.252
Male gender	6.724	0.801–56.432	0.079
Nucleosomes (AU)	33.070	1.216–899.485	0.038
CRP (mg/L)	1.000	0.993–1.006	0.883
Creatinine (μmol/L)	1.017	0.998–1.036	0.088
Nucleosomes ≥0.39 AU	5.467	1.051–28.432	0.043
CRP ≥ 227 mg/L	1.667	0.271–10.244	0.581
Creatinine ≥139 μmol/L	7.167	0.598–85.946	0.120
Multivariate analysis			
Male gender	6.048	0.693–52.799	0.104
Nucleosomes (AU)	33.070	1.216–899.485	0.038

*AU* absorbance unit, *CRP* C-reactive protein, *OR* odds ratio

## Discussion

The results show that the circulating nucleosome levels in patients with AP are elevated, associate with the severity of AP and predict, on admission to hospital, the development of severe AP among the patients who present without clinical signs of OD (MMS <2). Our results are in accordance with the finding that circulating DNA levels are elevated in patients with severe AP [31, 32] and that nucleosome levels are elevated in experimental pancreatitis [24]. To our knowledge, this study demonstrates, for the first time, the predictive value of circulating nucleosomes in AP.

Several biomarkers have been evaluated as predictors of the course of AP [33–37]. However, in these studies, OD group consistently comprised all patients with OD, in other words, the patients who have OD already at presentation and the patients who present without OD but are bound to develop it. Including the former may distort the results. Accordingly, in the present study, the analysis of all OD patients revealed that nucleosome levels predict OD; the analysis confined to patients with moderately severe and severe AP, excluding mild AP, showed that nucleosome levels did not predict OD, while only the nucleosome levels proved to predict OD among the patients presenting without OD. To our knowledge, nucleosomes, as demonstrated in the present study, and the adenosine-generating ecto-5′-nucleotidase/CD73 [11] and the cytokines interleukin 8, hepatocyte growth factor, and granulocyte-colony stimulating factor [12], are so far the only markers that may aid to identify the patients who present without signs of OD but are bound to develop it during the course of AP.

In the present study, we used highly specific cutoff values (>90 %) instead of maximizing the sum of sensitivity and specificity. The former setting, resulting in low sensitivity of the markers, is, we think, more real in the clinical work with limited ICU capacity. With the maximized sum of sensitivity and specificity (Figs. 2a and 2c), the sensitivity of circulating nucleosomes in predicting OD would have reached 83 % in the whole patient population (specificity 72 %) and 71 % among the patients without OD on admission (specificity 79.5 %).

The analysis of moderately severe AP and severe AP patients was performed, because mild AP patients may distort the results in the whole patient population, since they form the majority of AP patients and most of them recover uneventfully [11]. When the patients with mild AP were excluded from the analysis, we could not identify the patients with severe AP from those with moderately severe AP with any of the markers analyzed. In the analysis of patients who presented with OD (admission MMS ≥2), we tried to reveal if it was possible to predict the persistence of OD already on admission using circulating nucleosome, CRP, or creatinine levels. However, no difference between transient or persistent OD group was found (Table 3).

The finding may be explained, at least in part, by the origins of circulating nucleosomes in AP, which are not known in detail but are likely to be diverse. Neutrophils are an intriguing possibility as they are the most abundant leukocytes, are activated in patients with severe AP [38], and upon stimulation with cytokines, make extracellular traps [17] comprising DNA and core histone. Other sources of nucleosomes at least in experimental AP include apoptosis and necroptosis [39, 40] and tissue injury associated with circulatory shock/hypoperfusion [41]. As to the clinical point of view, it is impossible to say if the quickly, within 48 h, resolving OD is due to intensive treatment or represents the natural course of AP. Therefore, if the patient presents with OD, optimal treatment of severe AP needs to be started immediately, preferably in the ICU [29, 30].

The possibility that impaired renal function would contribute significantly to the increased nucleosome levels is not evident because nucleosome clearance appears to be mediated mostly by the liver [42–44]. In the present study, the major finding was that nucleosome levels predict the development of OD among the 14 patients who presented without OD. Creatinine levels of the patients were ≤170 μmol/L, as defined by MMS criteria [1]. Consequently, the predictive value of nucleosomes may not be explained by impaired renal function.

Identifying the patients who present without OD (MMS < 2) but are bound to develop severe AP is a great clinical challenge. Indeed, such patients form about half of the AP patients with OD [8, 11, 12]. The findings in the present study suggest that the on-admission levels of circulating nucleosomes aid to identify, on admission to hospital, the patients who present without OD but are bound to develop it. Among such patients, the levels of creatinine or CRP did not predict the development of severe AP in the present study or our previous studies [11, 12]. The present study, however, has limitations. The number of OD patients studied was limited, and the cutoff values were optimized. In addition, the storage time of the plasma samples was up to 10 years. Long-term stability investigations have revealed a 7 % decrease per year in serum levels of nucleosomes during sample storage at −70° [45]. However, the differences in the sample storage time may not explain our findings because the storage time did not correlate with nucleosome levels, and, furthermore, nucleosome level was an independent predictor of OD regardless of the sample age.

The release of DAMPs is considered to play central role in the pathogenesis of AP linking local tissue damage and death to systemic inflammatory response. Therefore, DAMPs might offer several novel therapeutic strategies in AP, such as preventing DAMP release [14], neutralizing or blocking DAMPs [46], or blocking the DAMP receptors or their signaling [47, 48]. The novel therapeutic modalities may be beneficial for the AP patients who present with OD, and, in particular, for the patients who present without OD but are bound to develop it.

## Conclusions

Our results show that the on-admission levels of circulating nucleosomes are elevated in AP and associated with the severity of the disease. In addition, our data show, for the first time, that nucleosome levels may serve as an independent predictor of severe AP among the patients who present without signs of OD (MMS < 2), the patient group which may be an optimal target for immunomodulatory treatment modalities.

## Abbreviations

AP: acute pancreatitis
APACHE II: acute physiology and chronic health evaluation II
AU: absorbance unit
AUC: area under the curve
CRP: C-reactive protein
DOR: diagnostic odds ratio
IQR: interquartile range
LR: likelihood ratio
MMS: modified marshall score
nDAMPs: nuclear damage-associated molecular patterns
NET: neutrophil extracellular traps
OD: organ dysfunction
ROC: receiver-operating characteristic
SOFA: sepsis-related organ failure assessment score
